# The microbiome quality control project: baseline study design and future directions

**DOI:** 10.1186/s13059-015-0841-8

**Published:** 2015-12-09

**Authors:** Rashmi Sinha, Christian C. Abnet, Owen White, Rob Knight, Curtis Huttenhower

**Affiliations:** Nutritional Epidemiology Branch, Division of Cancer Epidemiology and Genetics, National Cancer Institute, National Institutes of Health, 9609 Medical Center Drive, Bethesda, Maryland 20892 USA; Institute for Genome Sciences, University of Maryland School of Medicine, Baltimore, MD 21201 USA; Departments of Pediatrics and Computer Science & Engineering, University of California San Diego, La Jolla, CA 92093 USA; Department of Biostatistics, Harvard School of Public Health, Boston, MA 02115 USA

## Abstract

Microbiome research has grown exponentially over the past several years, but studies have been difficult to reproduce across investigations. Relevant variation in measurements between laboratories, from a variety of sources, has not been systematically assessed. This is coupled with a growing concern in the scientific community about the lack of reproducibility in biomedical research. The Microbiome Quality Control project (MBQC) was initiated to identify sources of variation in microbiome studies, to quantify their magnitudes, and to assess the design and utility of different positive and negative control strategies. Here we report on the first MBQC baseline study project and workshop.

## Challenges faced by the microbiome field

The efficient characterization of the microbial ecology of different human body sites using high-throughput sequencing has the potential to revolutionize research in chronic disease etiology. However, significant methodological challenges must be overcome to allow widespread application of these exciting new technologies. Two major initiatives, the Human Microbiome Project (HMP) [1] and MetaHIT [2], were launched to generate resources characterizing the microbiome in health and disease and to investigate the genes of the human intestinal microbiota, respectively. These initiatives included hundreds of individuals, and even with the associated methodology development, few subsequent epidemiological studies have evaluated the role of the microbiome in population health. The degree of standardization necessary for translation to large-scale studies is still in its infancy. It is crucial that experts from myriad specialist areas - clinicians, epidemiologists, microbiologists, ecologists, immunologists, statisticians, and bioinformaticians - work together to develop best practices and to identify sources of potential measurement variability through rigorous studies.

Addressing the sources of variation in microbiota profiling is critical for optimizing protocols and for understanding how best to combine different studies in a single pooled analysis. Unfortunately, variation at each step in the pipeline is enormous, from physical specimen collection and processing to computational quantification of microbial communities. The research community has not yet characterized which of these variations in protocols, both experimental and computational, may overwhelm biological effects of interest. Consequently, appreciation of the sources of variation in techniques, and of their relative magnitudes, must be assessed systematically.

Multi-center studies that assess technical variation among identical specimen replicates, across laboratories, and among protocols at each step in the pipeline, would greatly inform the field. Variation may arise from sample collection and storage methods, DNA extraction, PCR (for amplicon studies), DNA sequencing, bioinformatics, and statistical analyses. Large epidemiological studies of the human microbiome require the replication of findings across multiple populations, and pooling of data from many different cohorts, which, in practice, will include samples collected and processed at different times. To advance this field, it is essential to provide a palette of appropriate standards for each step of the assay process.

## The MBQC project

The Microbiome Quality Control project, or MBQC, is a collaborative effort designed to comprehensively evaluate methods for measuring the human microbiome. The project was inspired by earlier work in transcriptomics, for example, the Microarray Quality Control project (MAQC) [3], and we hope to improve the state-of-the-science in microbial community sample collection, DNA extraction, sequencing, bioinformatics, and analyses, while promoting open sharing of standard operating procedures and best practices throughout the field. For the baseline phase of the MBQC, we focused on comprehensively evaluating methods for amplicon profiling of the human fecal microbiome, including the techniques and protocols for handling microbiome samples and computational pipelines for microbial data processing.

## Standard reference materials

At the time we planned the MBQC baseline, no standard reference materials for positive and negative controls in microbiome analysis (such as a National Institute of Standards and Technology (NIST) standard reference material) were available. We included two positive control standards in the project. First, we created a bulk quality control (QC) sample that could be used throughout the MBQC-base and in future studies. The laboratory of Dr. Emma Allen-Vercoe at the University of Guelph, Canada, created a large quantity of fecal derived material grown in a chemostat bioreactor (referred to as the ‘bench-top colon’ or ‘Robogut’ [4]) that supports the growth of anaerobes and can create liters of homogenous material. This gave us material of identical composition exceeding that provided by even the most generous stool donor. These chemostat samples allowed us to assess whether DNA extraction, amplification, or sequencing choices leads to differences in microbiome measurements when using an identical, controlled source. Routine inclusion of comparable standard samples in future studies will allow a single laboratory to assess their own performance over time.

This chemostat positive control alone, however, has several important limitations. First, although a large volume can be produced, no one batch is limitless, and it cannot be identically reproduced when the supply is exhausted. Second, the full breadth of microbes possible across the human population cannot be represented in a single stool samples, and therefore a single sample will not allow the detection of taxon-specific artifacts induced by specific protocol changes. Third, we have no gold standard measurement of the exact list of organisms present in this sample or their true relative abundances.

To address this final limitation, we created an additional positive control samples that we refer to as ‘artificial colonies’ or mock communities: one comprised of 20 species known to inhabit human stool and a second comprised of 22 species from the human oral cavity. All species were selected from HMP reference strains that were originally isolated from humans. Each strain was grown as a single culture and then mixed using a microbiological loop at fixed ratios (most at a 1:1 ratio, but some at lower relative abundance). These specimens provide ground truths and allow us to assess the validity of a measurement method. To verify that our samples contained only the 20 or 22 intended species, we completed shotgun sequencing of these samples. The artificial colonies also allow us to analyze the 16S rRNA amplicon sequencing results directly by providing the exact 16S rRNA gene sequences present in the samples.

## MBQC study design

We used four types of samples in the MBQC: (1) 11 unique fresh stool samples; (2) seven unique freeze-dried stool samples; (3) two unique chemostat samples generated from a Robogut; and (4) two artificial colonies representing the gut and oral cavity. DNA from these four types of samples was extracted at one central laboratory. Duplicates and triplicates of both original and pre-extracted samples were combined to produce a 96-element sample set. This final set included 41 aliquots of centrally extracted DNA, 53 aliquots of the raw samples (frozen and freeze dried feces, chemostat, and artificial colony), and two negative control aliquots of storage buffer (10 mM Tris–HCl, pH 8.5). Participating labs were blinded to the identities of the samples.

Groups participating in the MBQC took part in either or both of handling samples for data generation and/or bioinformatics processing of the resulting data. Handling laboratories committed to extracting DNA from raw samples, amplifying 16S rRNA gene fragments and sequencing using either the Illumina MiSeq or HiSeq platform, and providing the resulting data to the wider group. To participate in the bioinformatics module, laboratories were asked to take re-blinded, demultiplexed Illumina FASTQ files and provide one Operational Taxonomic Unit (OTU) table and one phylogenetic tree as the final output (although labs were able to provide multiple final outputs, including, for example, closed-reference OTU tables defined by database match or open-reference or *de novo* OTU tables identifying new taxa).

Sixteen laboratories participated in the handling aspect and nine laboratories participated in the bioinformatics module of the MBQC.

Each of the handling laboratories independently chose protocols for DNA extraction, PCR amplification, and sequencing, which were then recorded in standardized templates at a high level of detail. This resulted in data from seven different DNA extraction kits and two different 16S hypervariable region primers (V4 515 F/806R [5, 6] and V3-4 318 F/806R [7]), and while the majority of labs (12 of 16) reported using homogenization during DNA extraction, not all labs included this step. All laboratories save one generated data using the Illumina MiSeq platform, and the final laboratory used a HiSeq 2500, most (14 of 16) in paired end mode. The resulting read lengths varied between 150 to 300 nucleotides, with PhiX control percentages spanning a wide range from none to 30 %. Ultimately, raw data comprised some 155 million total reads from 2,238 samples, including replicate sequencing from some groups, and a span from 57 % (on HiSeq) to 96 % quality bases per sample.

Bioinformatics participants also each used their own protocols of choice, which were again subsequently recorded. Typical bioinformatic steps included removal of low-quality bases or shortened reads (for example, by Trimmomatic [8]), paired read stitching (for example, by PANDAseq [9]), OTU construction (for example, by QIIME [10] or UPARSE [11]), and quality control of the resulting table. A total of 16,555 samples were analyzed after incorporating replicates from all nine labs, and the final OTU tables were assessed to identify the effects of wet- and dry-lab protocol variables on within-sample (for example, alpha-diversity and clade abundances) and between-sample (for example, beta-diversity) analyses.

## Preliminary results

The scope of the MBQC baseline is of necessity preliminary, given the limited funding available with which to survey only a few sample types, data generation strategies, and computational pipelines, primarily by much-appreciated volunteer effort. However, two encouraging initial conclusions can be drawn from the project’s surprisingly extensive data, which comprised over 155 million sequence reads and some 16,555 fully analyzed samples, more than the Human Microbiome Project itself. First, a relatively small number of laboratory protocol and bioinformatics variables accounted for the majority of detected variability; many potential sources of variation, such as sequencing platform or error rate, proved to have at most very small effects. Second, these sources of technical variability were generally of the same order of magnitude as those induced by biological phenotypes, suggesting that they can be overcome in most studies by careful adherence to standardized protocols. However, the complex patterns of differences between analyzed samples ultimately suggested complex interactions between sample, extraction, and bioinformatics in terms of arriving at an overall profile of the microbes in a given sample.

Experimentally, DNA extraction method was a major source of variation, although in the baseline study design this was largely confounded with the lab performing the analysis. This represents one of several additional study design elements to be incorporated into a full-scale MBQC, with each DNA extraction kit tested in multiple labs. The choice of 16S amplification primer produced as large or larger an effect on generated data, although again only a few such primers were evaluated during the baseline, and sequencing depth and sample storage and transportation both had relatively small but detectable effects on microbiome data generation.

Positive and negative controls, in the form of mock communities (of known composition) and samples containing no DNA, were particularly informative for quality control. Approximately half of sample handling labs produced a non-trivial number of reads for negative control samples, although this was likely due mainly to differences in sample exclusion between labs (due to low DNA concentration). Some bioinformatics protocols were able to reduce the number of false positive OTUs in these samples, but in most cases when a negative control sample was sequenced, it resulted in up to several hundred putative OTUs. Positive controls likewise were often analyzed as containing between 50 and 150 OTUs, instead of the target 20. These spurious OTUs were typically in the correct phylogenetic areas, as indicated by low UniFrac distances to the known true composition, with the main variation in accuracy in this case again arising from wet- rather than dry-lab protocol choices. Intriguingly, many of these spurious OTUs may be due to bleed-through from other samples sequenced simultaneously, since they were generally derived from gut-resident microbes seen at much higher abundance in the fecal samples in adjacent wells.

Different bioinformatics pipelines varied greatly in putative OTU table composition and in their absolute alpha- and beta-diversity estimates, but similar patterns appeared across samples with almost all pipelines. Quality control choices were among the largest sources of variability among bioinformatics protocols, both at the level of raw sequences (for example, quality trimming, filtering, and stitching) and at the level of feature (that is, OTU) or sample removal from the final table. Different choices of OTU construction strategies, ranging from unsupervised clustering to direct classification, also induced smaller differences in inferred taxonomic composition. Ultimately, reproducibility underlies the replication of results needed to draw inferences regarding health and disease for epidemiologic studies, making appropriate standardized bioinformatics protocols as or more crucial than experimental procedures for consistency over time and laboratory, especially for longitudinal studies.

All data from the MBQC baseline study are available from the SRA under BioProject ID SRP047083.

## MBQC baseline conclusions

There was a consensus across the MBQC participating groups that although microbiome measurements face substantial challenges, this baseline project was very successful and represented impressive progress towards the overall goal of establishing practical guidelines for reproducibility within labs over time and across the field. The benefits to maximizing reproducibility are clear: overall increase in scientific rigor, reduction of redundant effort from researchers developing their own methods, improving the meaningfulness of comparing results from independent studies, and increasing power and sample size by meta-analytic combinations of results from multiple studies.

## Aims of the consortium for the future

For the future, it would be useful to expand the MBQC project in several directions. These include: (1) more diverse sample sources and collection methods; (2) developing further standard reference and control materials; (3) expanding the MBQC baseline study to include swapping samples between laboratories, replication of protocols among laboratories, and focusing on the details of protocol variables highlighted by the baseline (for example, extraction and PCR); (4) defining the contaminant profiles of common reagents; (5) comprehensively evaluating variables using a Latin square or comparable spike-in design; and (6) open access teaching materials and workshops that would share these results allowing labs to define best practices in human microbiome studies.

1. Sample collection
  It is important to evaluate and standardize sample collection protocols to reliably measure the human microbiome in large-scale population studies. Best practices for collections of microbiome samples from different anatomic sites (for example, gut, cervix, oral cavity, and skin) must be considered in the context of overall study designs. For example, particularly stringent collection protocols for clinic-based studies are not feasible for large epidemiologic research in which acceptability by participant is paramount and controlling cost is essential. To expand human microbiome research into the population sciences for etiologic research, several issues are pertinent. First, the method of collection must preserve the microbial signature for each sample. Second, collection and storage conditions must render the biological sample stable under field conditions over an extended period of time in less than optimal storage conditions. Third, samples collected in a prospective study should be preserved in a way that maximizes its potential for use with multiple platforms (for example, microbiomics, metabolomics, transcriptomics). Finally, future epidemiologic studies of the microbiome will likely need to be very large to adjust for multiple comparisons and data may need to be pooled or meta-analyzed from multiple studies from different laboratories.
  Further questions will need to be considered in evaluating sample collection for diverse populations, body sites, study purposes, and epidemiological contexts. It is important to reflect on whether a portion of the sample represents the ‘whole’ of the site-specific microbiome of an individual or whether there is need to collect multiple aliquots from the same sample (for example, the degree to which within-sample biogeography matters for a stool sample). In some contexts, a one-time collection may be adequate, while in others multiple collections from individuals over time may be necessary. Different sampling devices and collection media are available and need to be evaluated side-by-side for both ease of use and integrity of sample. Storage at −80 °C is a standard protocol, but the impact of long-term archival storage and freezing and thawing of samples on the integrity of microbiome is still an open question. In summary, more work is required to develop standardized sample collection protocol for field studies that will allow reproducible and valid characterization of the microbiome.
2. Ideal standard reference materials
  Positive and negative controls are vital for any experiment, and their adoption through standard reference materials in the context of human microbiome studies is still in flux. The MBQC used two types of standards, produced in a chemostat and artificial colonies, but neither constitutes a sufficient standard reference material for all microbiome research. Ideally, positive control reference materials would include curated taxa representing many body habitats, or be site- or population-specific to mimic a ‘typical’ microbial community for broad classes of studies. Considerations for such controls include taxa with a variety of GC contents, mixed in widely staggered ratios, and with absolute quantitation of the number of cells and/or genomes included. Negative control requirements are equally critical, including blanks for each kit or preparation reagent used during sample handling, and in some cases within- and between-batch spatial or temporal replication as well. Both types of controls will improve our ability to normalize across batches and between experiments. Producing such types of standards requires the concerted efforts of the community and of experienced organizations such as the National Institute of Standards and Technology.
3. Expand the MBQC baseline study
  Although the MBQC baseline has yielded scientifically interesting and technically significant results, far more remains to be done to scale human microbiome research over the next years and decades. Only 16S rRNA amplicon analyses were performed during the baseline study, leaving extension of the results to other ‘omics techniques as an especially valuable next step, as similar questions apply to metagenomics, metatranscriptomics, metaproteomics, and metabolomics. This iteration did not examine variability due to specimen collection or storage protocols, yet we know that both of these can have effect sizes that outweigh some of the more subtle biological effects in real samples and patients. While individual samples were replicated, per-subject biological replicates, longitudinal samples, and plate replicates were all insufficient to allow analysis of variability among these other replicate types. Similarly, the number of samples from a given biological condition was, in general, too low for detailed interpretation of which types of variation among humans could be detected against a background of methodological variation. Consequently, an expanded MBQC that addresses these important parameters could be of tremendous value to the field.
  Additionally, although we observed substantial variation due to DNA extraction and sequencing protocols, the features of non-standard protocols were largely unique to individual laboratories, making it impossible to isolate specific effects of individual steps. This was of course not by design, but due to the need to impose minimal proscriptive restrictions on groups participating solely on a volunteer basis and it helped us enumerate how different labs implement their analyses. A future study that runs the same set of varied protocols in each of a number of labs would provide considerable additional insight into which steps are critical to reproducible results and which steps can be varied without consequence, allowing the most attention to be paid to the most important steps in the pipeline. Swapping unextracted samples among laboratories as well as swapping DNA among laboratories would also help characterize these potential issues.
4. Define more reliable reagents
  As microbiome studies move to lower input microbial biomass, identifying and eliminating contaminants in reagents that lead to incorrect detection of non-biologically relevant taxa is increasingly important. The blank wells throughout MBQC often yielded some sequences, allowing insight into these contaminants, although the scope of the baseline project did not allow for testing reagent decontamination protocols. The baseline also included only simple buffer blank controls, whereas while carrying out diverse experimental protocols, each kit or reagent can introduce unique contaminants, sometimes varying in their abundances by orders of magnitude and able to swamp true biological signal from low-biomass samples. A more detailed analysis of reagent contamination and countermeasures to reduce it could therefore be of substantial value to the field.
5. A comprehensive Latin square spike-in design
  The results from the MBQC synthetic communities highlight the need for additional studies of the effects of DNA extraction and PCR on the ability to accurately and precisely detect organisms at different levels of abundance. This task is difficult because detection limits may depend on specific sequences as well as the underlying organismal concentration, let alone different bioinformatics strategies. One method of addressing this issue is to use a Latin square (or comparable) design, in which each protocol variable is modified at least once in the hands of each different lab. This is easiest in a setting where synthetic communities are spiked into a background of replicate samples, allowing readout of the known members of the community in the context of a complex background.
  Challenges to this design include the need to standardize protocols across many labs, which was impossible in the limited resource setting of the baseline study. Additionally, it can be difficult to construct synthetic communities of measurably well-defined composition because of issues including variation in copy number of the 16S rRNA gene, DNA to biomass ratio, and so on. However, expanded sets of spike-in controls of known composition would substantially aid in quantifying accuracy, in addition to variability, induced by each protocol variable choice across labs. The Latin squares design can be extended when feasible to any protocol variables in the scope of the MBQC, experimental or computational, allowing both the accuracy of taxonomic assessment and variability from other protocol choices to be evaluated systematically.
6. Teaching materials and workshops
  Finally, there is tremendous demand in academia, government, industry, and healthcare for workers trained in robust microbiome analyses. This need of course extends well beyond the scope of the MBQC alone, but teaching materials based on the MBQC experience, especially those aimed at describing robust and reproducible analyses and at identifying common pitfalls that can lead to incorrect results, should be developed. Coupled with community outreach and the incorporation of diverse researcher feedback into future iterations of the project, we anticipate that the standardization, open access data generation, protocol sharing, and facilitation activities of future expansions of the MBQC will be instrumental in translating human microbiome studies into an effective understanding of population-level microbial community biology and public health.

## References

[1] Human Microbiome Project Consortium (2012). Structure, function and diversity of the healthy human microbiome. Nature.

[2] Qin J (2010). A human gut microbial gene catalogue established by metagenomic sequencing. Nature.

[3] Consortium M, Shi L, Reid LH, Jones WD, Shippy R, Warrington JA (2006). The MicroArray Quality Control (MAQC) project shows inter- and intraplatform reproducibility of gene expression measurements. Nat Biotechnol.

[4] McDonald JA, Fuentes S, Schroeter K, Heikamp-deJong I, Khursigara CM, de Vos WM (2015). Simulating distal gut mucosal and luminal communities using packed-column biofilm reactors and an in vitro chemostat model. J Microbiol Methods.

[5] Caporaso JG, Lauber CL, Walters WA, Berg-Lyons D, Huntley J, Fierer N (2012). Ultra-high-throughput microbial community analysis on the Illumina HiSeq and MiSeq platforms. ISME J.

[6] Kozich JJ, Westcott SL, Baxter NT, Highlander SK, Schloss PD (2013). Development of a dual-index sequencing strategy and curation pipeline for analyzing amplicon sequence data on the MiSeq Illumina sequencing platform. Appl Environ Microbiol.

[7] Fadrosh DW, Ma B, Gajer P, Sengamalay N, Ott S, Brotman RM (2014). An improved dual-indexing approach for multiplexed 16S rRNA gene sequencing on the Illumina MiSeq platform. Microbiome.

[8] Bolger AM, Lohse M, Usadel B (2014). Trimmomatic: a flexible trimmer for Illumina sequence data. Bioinformatics.

[9] Masella AP, Bartram AK, Truszkowski JM, Brown DG, Neufeld JD (2012). PANDAseq: paired-end assembler for illumina sequences. BMC Bioinformatics.

[10] Caporaso JG, Kuczynski J, Stombaugh J, Bittinger K, Bushman FD, Costello EK (2010). QIIME allows analysis of high-throughput community sequencing data. Nat Methods.

[11] Edgar RC (2013). UPARSE: highly accurate OTU sequences from microbial amplicon reads. Nat Methods.

